# Effect of Dickkopf-1 (Dkk-1) and SP600125, a JNK Inhibitor, on Wnt Signaling in Canine Prostate Cancer Growth and Bone Metastases

**DOI:** 10.3390/vetsci8080153

**Published:** 2021-08-01

**Authors:** Wachiraphan Supsavhad, Bardes B. Hassan, Jessica K. Simmons, Wessel P. Dirksen, Said M. Elshafae, Nicole A. Kohart, Aylin A. Demirer, Thomas J. Rosol

**Affiliations:** 1 Department of Veterinary Biosciences, College of Veterinary Medicine, The Ohio State University, Columbus, OH 43210, USA; wachiraphan.s@ku.th (W.S.); dr_bardis@cu.edu.eg (B.B.H.); jsimmons@seagen.com (J.K.S.); dirksen.8@osu.edu (W.P.D.); saidelshafae@yahoo.com (S.M.E.); nicolekcromwell@gmail.com (N.A.K.); aylindemirer@gmail.com (A.A.D.); 2 Department of Pathology, Faculty of Veterinary Medicine, Kasetsart University, Bangkok 10900, Thailand; 3 Department of Pathology, Faculty of Veterinary Medicine, Cairo University, Giza 12211, Egypt; 4 Department of Neuroscience and Pharmacology, University of Iowa, Iowa, IA 52242, USA; 5 Department of Pathology, Faculty of Veterinary Medicine, Benha University, Kalyubia 13736, Egypt; 6 Pathology Department, Faculty of Veterinary Medicine, Bursa Uludag University, Bursa 16059, Turkey; 7 Department of Biomedical Sciences, Heritage College of Osteopathic Medicine, Ohio University, Athens, OH 45701, USA

**Keywords:** prostate cancer, SP600125, bone metastasis, Wnt signaling, JNK inhibitor, dog

## Abstract

Human Dickkopf-1 (Dkk-1) upregulates a noncanonical Wnt/JNK pathway, resulting in osteoclast stimulation, cell proliferation, and epithelial-to-mesenchymal transition (EMT) of cancer cells. Ace-1-Dkk-1, a canine prostate cancer (PCa) cell line overexpressing Dkk-1, was used to investigate Wnt signaling pathways in PCa tumor growth. SP600125, a JNK inhibitor, was used to examine whether it would decrease tumor growth and bone tumor phenotype in canine PCa cells in vitro and in vivo. Ace-1-Vector^YFP-Luc^ and Ace-1-Dkk-1^YFP-Luc^ cells were transplanted subcutaneously, while Ace-1-Dkk-1^YFP-Luc^ was transplanted intratibially into nude mice. The effects of Dkk-1 and SP600125 on cell proliferation, in vivo tumor growth, and bone tumor phenotype were investigated. The mRNA expression levels of Wnt/JNK-related genes were measured using RT-qPCR. Dkk-1 significantly increased the mRNA expression of Wnt/JNK-signaling-related genes. SP600125 significantly upregulated the mRNA expression of osteoblast differentiation genes and downregulated osteoclastic-bone-lysis-related genes in vitro. SP600125 significantly decreased tumor volume and induced spindle-shaped tumor cells in vivo. Mice bearing intratibial tumors had increased radiographic density of the intramedullary new bone, large foci of osteolysis, and increased cortical lysis with abundant periosteal new bone formation. Finally, SP600125 has the potential to serve as an alternative adjuvant therapy in some early-stage PCa patients, especially those with high Dkk-1 expression.

## 1. Introduction

Prostate cancer (PCa) is the second most common cancer in men, with 248,530 newly diagnosed cases and 34,130 deaths in 2021 in the United States [1]. The incidence of this cancer increases with age. The majority of patients with advanced PCa die from metastatic disease, where bone is the most common metastatic site. PCa bone metastases are predominantly osteoblastic (new bone formation) [2]. Metastasis to bone is associated with nerve compression, severe pain, and decreased quality of life [3]. Even though studies have shown connections between bone metastasis and the bone microenvironment in prostate cancer, there are many details that need to be clarified.

Several factors are involved in PCa-induced osteogenesis, including Wnt proteins [2,4]. Wnt signaling is classified into two major pathways: canonical (β-catenin-dependent) and noncanonical (β-catenin-independent) pathways. The canonical pathway is an important pathway in bone microenvironment regulation, new bone formation, osteoblast differentiation [5,6], and osteoclastogenesis suppression through upregulated expression of osteoprotegerin (OPG) and downregulated expression of the receptor activator of nuclear factor-κB ligand (RANKL) [7,8]. The noncanonical pathway is classified into two subpathways: the Wnt/Ca^2+^ pathway and the Wnt/planar cell polarity (Wnt/JNK) pathway [9,10].

The Wnt family consists of 19 mammalian Wnts that bind to transmembrane receptors termed Frizzled (FZD) to activate the canonical and noncanonical pathways. There are 10 FZD receptors that have been identified in mammals [11]. In the canonical pathway, Wnt proteins form a complex (WNT/FZD/LRP5/6) with the transmembrane receptors FZD and the coreceptor low-density lipoprotein (LDL) receptor-related protein 5/6 (LRP5/6) to inhibit the degradation of β-catenin in the cytoplasm. The accumulated β-catenin then translocates to the nucleus, where it binds to T-cell factor (TCF)/lymphocyte enhancer factor (LEF) transcription factors and upregulates Wnt target genes that are needed for new bone formation [10]. In contrast, the role of noncanonical Wnt signaling in bone-metastatic PCa is unclear. In this pathway, Wnt proteins transduce their signal independently from LRP5/6 to activate c-Jun N-terminal kinase (JNK) and its downstream targets [11]. A correlation between high levels of JNK-associated proteins and osteoblast malignant transformation as well as tumor progression was reported in osteosarcoma [12]. JNK has been implicated in transcription factor activation, mRNA stabilization, apoptosis regulation, and PCa progression [13,14]. Moreover, JNK upregulation is associated with resistance to chemotherapeutic drugs [15]. SP600125, an anthrapyrazolone and a small-molecule inhibitor of the JNK catalytic activity, has been reported to reduce tumor cell growth and invasion and induce apoptosis [13,16].

Activator protein-1 (AP-1) is involved in PCa development and progression by regulating the expression of genes implicated in proliferation, apoptosis, angiogenesis, and cancer cell invasion and metastasis [17,18]. AP-1 transcription factors are formed by either Jun-Jun homodimers or Jun-Fos heterodimers [19]. c-Jun and c-Fos were upregulated in a mouse model of human PCa progression, and their upregulation was associated with PCa proliferation and invasion [20].

Dickkopf-1 (Dkk-1) is a secreted glycoprotein that acts as both a potent inhibitor of canonical Wnt signaling and an upregulator of the noncanonical Wnt/JNK signaling pathway [21,22]. Dkk-1 is one of the osteoblastogenesis (new bone formation) inhibitors, which blocks the canonical Wnt pathway by binding to LRP5/6 and inhibiting WNT/FZD/LRP5/6 complex formation [21]. High levels of Dkk-1 were commonly detected in early-stage PCa and decreased after PCa progression with bone metastases. The decreased Dkk-1 enhanced osteogenesis by Wnt and altered the phenotype of bone metastases from osteolytic to osteoblastic [2]. Moreover, early blockage of Dkk-1 expression in PCa metastasis prevented tumor development in the bone, suggesting that osteolysis is a critical first step in the progression of PCa bone metastases [23]. The association between high-serum Dkk-1 and short survival time in PCa patients was previously reported [24]. Moreover, serum Dkk-1 level was useful in the prognosis of patients with a high risk of PCa development and a previous negative biopsy [25]. A study on canine PCa demonstrated that alteration of Dkk-1 expression in a dog PCa cell line (Ace-1/DKK-1^YFP-LUC^) changed the metastatic phenotype and tumor growth in vivo [4]. However, the role of Dkk-1 in canonical and noncanonical Wnt signaling pathways in canine PCa remained unclear.

In this study, we hypothesized that Dkk-1 would inhibit canonical Wnt signaling and activate the noncanonical Wnt/JNK pathway in canine PCa. Moreover, inhibiting noncanonical Wnt/JNK signaling using a JNK inhibitor (SP600125) would induce canonical signaling in Ace-1-Dkk-1 and decrease tumor growth and the bone metastatic phenotype of PCa. To test these hypotheses, Ace-1-Dkk-1, a canine PCa overexpressing human Dkk-1 previously developed in our laboratory, was used to elucidate the molecular role of Wnt signaling and Dkk-1 in canine PCa growth and bone tumor phenotype and growth.

## 2. Materials and Methods

### 2.1. Cell Culture

Ace-1-Dkk-1, a dog prostate cancer cell line stably transfected with human Dkk-1, and Ace-1-Vector (empty vector) were previously developed by our laboratory [4]. Ace-Dkk-1^YFP-Luc^ and Ace-Vector^YFP-Luc^ were stably transfected with firefly luciferase, as described previously [4]. These cells were grown in Dulbecco’s Modified Eagle’s Medium/Ham’s Nutrient Mixture F12 (DMEM/F12) (Invitrogen, Carlsbad, CA, USA) supplemented with 10% fetal bovine serum (FBS) (Sigma-Aldrich Co., St. Louis, MO, USA) and 1% penicillin/streptomycin (Life Technologies, Grand Island, NY, USA) at 37 °C in a 5% CO_2_-humidified environment.

### 2.2. Dkk-1 ELISA

Ace-1-Vector and Ace-1-Dkk-1 cells were seeded in 6-well culture plates in triplicate at a density of 500,000 cells per well and cultured in DMEM/F12 with 10% FBS and 1% penicillin/streptomycin. After 24 h, medium was collected, and Dkk-1 concentrations were measured using the DuoSet Human Dkk-1 ELISA Kit (R&D Systems, Minneapolis, MN, USA) [26]. The lowest standard of the assay was 62.5 pg/mL.

### 2.3. Immunohistochemistry

Ace-1-Vector and Ace-1-Dkk-1 were grown to 70% confluency, trypsinized, and centrifuged at 1000 RPM for 5 min. The cell pellet was then fixed in 10% neutral-buffered formalin for 24 h and embedded in paraffin. The specimens were sectioned (5 μm). Then they were deparaffinized, rehydrated, and incubated in antigen retrieval solution (Dako, Carpinteria, CA, USA) at 90 °C for 30 min before cooling down at room temperature for 15 min. The endogenous peroxidase was blocked using 3% hydrogen peroxide and serum-free protein block (Dako). Samples were incubated at room temperature for 1 h with monoclonal mouse anti-β-catenin (BD Pharmingen, San Jose, CA, USA, Cat. No. 610153, 1:250) in phosphate-buffered saline (PBS). Anti-mouse monoclonal (Vector Laboratories, Burlingame, CA, USA, Cat. No. BA-9200, 1:200) in PBS was applied as the secondary antibody. Slides were stained with a Vector ABC Elite complex (Dako) for 30 min, diaminobenzidine tetrahydrochloride (Dako) for 5 min, and Mayer’s hematoxylin for 1 min. Image Pro Plus 9.0 (Media Cybernetics, Rockville, MD, USA) was used to perform batch analysis and quantify total cell staining (reported as optical density, OD). The values from 5 fields of approximately 700 cells were averaged.

### 2.4. Activator Protein-1 (AP-1) Reporter Transfection

To investigate the activity of Wnt/JNK signaling, 10,000 Ace-1-Vector or Ace-1-Dkk-1 cells in 96-well plates in triplicate were transiently transfected with 0.1 μg of either an inducible AP-1 reporter construct, a negative control, or a positive control purchased from SABiosciences (Qiagen, Germantown, MD, USA, Cat. No. CCS-011L). Transient transfection was accomplished using a 0.5 μL lipofectamine 2000 transfection reagent (Invitrogen, Waltham, MA, USA) and Opti-MEM media (Life Technologies, Carlsbad, CA, USA) according to the manufacturer’s instructions. Cells were cultured for 24 h in DMEM/F12 and 0.1% bovine serum albumin (BSA) alone or with 20 μM of SP600125 (Santa Cruz Biotechnology, Dallas, TX, USA), a selective JNK inhibitor. A constitutively active Renilla luciferase was used as an internal control to normalize for transfection efficiency. The negative control was used to normalize for background luciferase reporter activity.

### 2.5. Cell Proliferation Assay

Ace-1-Vector and Ace-1-Dkk-1 cells were seeded in a 6-well plate in triplicate at a density of 200,000 cells per well. Cells were cultured in DMEM/F12 with 0.1% BSA and incubated at 37 °C and in 5% CO_2_. The cells were collected using 0.25% trypsin at 24, 48, and 72 h after the initial plating and counted with a Cellometer automated T4 cell counter (Nexcelom Bioscience, Lawrence, MA, USA) using trypan blue dye exclusion to differentiate between live and dead cells. The doubling time was calculated using the formula (t2- t1) − log(n_2_)/log(n_2_/n_1_), where *n* is the cell number at given time points (t).

### 2.6. Wound Healing Assay

Cell migration was measured using a wound healing assay for Ace-1-Vector and Ace-1-Dkk-1 cells (control and SP600125 treated), which were grown to 100% confluence in 6-well plates in triplicate. Media were removed, cells were rinsed with sterile Dulbecco’s phosphate-buffered saline (DPBS), and a sterile 200 µL pipet tip was used to scratch 3 separate wounds through the cells. The cells were rinsed with DPBS to remove cell debris and cultured in 1.5 mL of DMEM/F12 with 0.1% BSA alone or with 20 μM SP600125 (Santa Cruz Biotechnology) and incubated at 37 °C and in 5% CO_2_. Using an inverted-phase contrast microscope with a digital camera, images of the scratches were photographed at 0, 6, 12, and 24 h. The rate of wound closure was calculated using the slope of the line graph created from the time point data.

### 2.7. JNK Inhibitor (SP600125) Treatment In Vitro

Three different passages of Ace-1-Vector and Ace-1-Dkk-1 cells were grown to 90% confluence in a 6-well plate in triplicate. Cells were then cultured for 24 h in DMEM/F12 and 10% FBS with either 20 μM SP600125 (Santa Cruz Biotechnology) or an equal amount of the carrier, 0.1% dimethyl sulfoxide (DMSO). After a 24 h incubation period, the cells were collected to measure the mRNA levels of bone-related genes by quantitative reverse transcription PCR (RT-qPCR).

### 2.8. RNA Extraction and Quantitative RT-PCR

Total RNA was extracted from the Ace-1-Vector and Ace-1-Dkk-1 cells treated with either 20 µM SP600125 or 0.1% DMSO using the RNA cultured cell HC kit (QuickGene by AutoGen, Holliston, MA, USA) according to the manufacturer’s protocol for adherent tissue culture cells. RNA was also extracted from the mice xenografts (Section 2.9) to assess the JNK-related gene expression. Total RNA (0.5 μg) was reverse-transcribed using SuperScript II (Invitrogen), and RT-qPCR was performed in duplicate using the QuantiTect SYBR Green PCR Kit (Qiagen, Germantown, MD, USA, Cat. No. 204145) and a LightCycler^®^ 480 (Roche, Indianapolis, IN, USA). PCR cycling conditions were as follows: 95 °C 15 min (preincubation), followed by 45 cycles of 94 °C/15 s, 56 °C/20 s, and 72 °C/10 s and a melting curve beginning at 65 °C and going up to 93 °C at a ramp rate of 0.11 °C/s with continuous fluorescence monitoring. Primer concentrations were 0.5 μM each, and 2 μL cDNA was added to each PCR reaction (20 μL). Every experiment/plate included no RT controls of all of the samples. The housekeeping gene, glyceraldehyde 3-phosphate dehydrogenase (*GAPDH*), and the following genes were quantified: bone morphogenetic protein-2, 4, and 7 (*BMP2*, *BMP4*, and *BMP7*); runt-related transcription factor 2 (*RUNX2*); receptor activator of nuclear factor kappa-B ligand (*RANKL*); osteoprotegerin (*OPG*); activating transcription factor 4 (*ATF4*); aldehyde dehydrogenase 1 family member 1 (*ALDH1A1*); Wnt family member 5A (*WNT5A*); Wnt family member 11 *(Wnt11)*; WW domain containing transcription regulator 1 (*WWTR1*); mitogen-activated protein kinase kinase 7 (*MAP2K7*); Jun proto-oncogene (*JUN)*; Fos proto-oncogene (*FOS)*; tumor protein p53 (*TP53)*; phosphatase and tensin homolog (*PTEN)*; phosphatidylinositol 3-kinase (*PIK3CA)*; *TWIST*; folate hydrolase 1 (*FOLH1*); transcription factor 4 (*TCF4*); mitogen-activated protein kinase 8 and 9 (*MAPK8* and *MAPK9*); serine/threonine kinase 1 (*AKT1)*; parathyroid hormone-related protein (*PTHrP)*; and transforming growth factor beta (*TGFβ)* using canine-specific primers (Supplementary Table S1). GAPDH was not differentially expressed between comparison groups. The RT-qPCR results were analyzed using the LightCycler^®^ 480 Software (Roche Life Science, Pleasanton, CA, USA), and relative mRNA expression was calculated using the delta-delta Ct (∆∆Ct) method: all values were normalized to their corresponding *GAPDH* values (∆Ct). All primers were designed using the Primer-BLAST software with standard melting temperatures (57−63 °C; Opt. 60 °C) and PCR product sizes (70–200 nt, or up to 250 nt, when necessary). We designed primer pairs that gave the smallest amplicons possible, while at the same time crossing the largest introns possible (or spanning an exon–exon junction, if the introns were small). The primers used in this study were chosen from 3 to 4 different primer pairs designed for each gene, and the primer pair that had the best amplification (slope) and RT-qPCR product melting (single peak) characteristics for each gene was chosen. The primer pairs were designed using the Primer-BLAST software (http://www.ncbi.nlm.nih.gov/tools/primer-blast; last accessed date: 31 August 2017). To confirm primer specificity, all RT-qPCR products were verified by electrophoresis on a 2% agarose gel and stained with ethidium bromide to confirm a single amplification product of the expected size. The entire PCR reactions were then purified using the QIAquick PCR Purification Kit (Qiagen, Cat. No. 28106) and sequenced at the Plant-Microbe Genomics Facility at OSU using a 3730 DNA Analyzer (Applied Biosystems, Grand Island, NY, USA) and BigDye Terminator Cycle Sequencing chemistry (Applied Biosystems). Sequences were verified by a BLAST search using the NCBI website (https://blast.ncbi.nlm.nih.gov/Blast.cgi; last access date: 31 August 2017). When checked, efficiencies have always been >95%, with most of them being 99%–100% efficient, and the Ct values obtained were in the linear range of amplification.

### 2.9. Subcutaneous Injection and JNK Inhibitor (SP600125) Treatment

Twenty-three 6-week-old athymic (NCR-nu/nu) male nude mice were purchased from the Ohio State University Comprehensive Cancer Center (OSUCCC) Target Validation Shared Resource (TVSR). Mice were subcutaneously transplanted with Ace-1-Dkk-1^YFP-LUC^ cells (*n* = 12) or Ace-1-Vector^YFP-LUC^ (*n* = 11) cells in equal number (1–2 × 10^7^ cells in 0.1 mL PBS). Treatment with SP600125 (Santa Cruz Biotechnology) or vehicle (2% DMSO, 2% polyethylene glycol 600 and 2% Tween 80 in PBS) was initiated when the tumor size reached 100–250 mm^3^ (treatment started on day 8 after injection). Mice bearing Ace-1-Dkk-1^YFP-LUC^ or Ace-1-Vector^YFP-LUC^ tumors were treated with intraperitoneal (IP) injection of an equal volume of either SP600125 or vehicle (5 mg/kg) every other day for 4 weeks [27]. Tumor size and body weight were measured every 2–3 days.

### 2.10. Intratibial Injection and JNK Inhibitor (SP600125) Treatment

Fifteen 6-week-old athymic (NCR-nu/nu) male nude mice were purchased from OSUCCC TVSR. All mice were injected with 5 × 10^5^ of Ace-1-Dkk-1^YFP-Luc^ cells, into the left tibial bone, as described [28]. The mice were treated (IP) with 5 mg/kg of an equal volume of either SP600125 (*n* = 8) or vehicle (*n* = 7) every other day for 2 weeks. The mice were euthanized on day 15 after transplantation.

### 2.11. Bioluminescent Imaging

Mice were intraperitoneally injected weekly with 0.1 mL of 40 mg/mL D-luciferin (Caliper Life Sciences, Hopkinton, MA, USA) dissolved in PBS prior to imaging. The mice were anesthetized in an induction chamber with a 3% isoflurane/oxygen mixture and maintained at 2% isoflurane using a nose-cone delivery system during imaging. Bioluminescent in vivo imaging was performed using IVIS 100 (Caliper Life Sciences), and photon signal intensity was quantified using the Living Image software, version 2.50 (Caliper Life Sciences). Imaging was performed every 2 min until peak photon signal was achieved (approximately 10 to 15 min postinjection). Bioluminescence was expressed as total photons/sec/region of interest.

### 2.12. Faxitron Imaging

On day 15 (euthanasia), radiographic images of the left tibia from each mouse were obtained using an LX-60 Faxitron laboratory radiography system (Faxitron X-ray Corp., Wheeling, IL, USA). The tibias were imaged at 28 KV for 5 s to qualitatively evaluate the effect of SP600125 on bone tumor growth in mice bearing Ace-1-Dkk-1^YFP-Luc^ intratibial tumors.

### 2.13. Histopathological Studies

The mice bearing subcutaneous tumors were euthanized after 4 weeks of treatment with either SP600125 or vehicle. Organs and subcutaneous xenografts were collected and examined to confirm metastases and tumor growth. All xenografts were weighed and divided into 2 halves: 1 half was immediately snap-frozen in liquid nitrogen and kept at −80 °C, and the other half was fixed in 10% natural-buffered formalin for 48 h, embedded in paraffin, sectioned, and stained with hematoxylin and eosin (HE).

The mice bearing intratibial tumors were euthanized at day 15 after they were transplanted with the tumor cells. Tibial bones were decalcified in a mild decalcifier (formaldehyde, methanol, and formic acid) (Leica Biosystems Inc., Buffalo Grove, IL, USA, 60089) at 37 °C for 4 h before histopathological processing. Images were acquired using an Olympus BX51 microscope (Olympus America, Inc., Melville, NY, USA, 11747) equipped with a Nikon digital camera (Nikon Inc., Melville, NY, USA, 11747).

### 2.14. Statistical Analysis

Data were analyzed using GraphPad PRISM 6.0 (GraphPad, San Diego, CA, USA). Results are displayed as means ± standard deviation. Normalized gene expression, AP-1 reporter activity, proliferation, and migration data were analyzed using a 2-way ANOVA, followed by Sidak’s multiple comparisons test to perform pairwise comparisons. The data from the Dkk-1 ELISA and β-catenin OD were analyzed by comparing the Ace-1-Dkk-1 group with the Ace-1-Vector group using Student’s *t*-test. The in vitro experiments were repeated in triplicate, and the values were expressed as the ratio between SP600125 treated and control groups (*y*-axis) ± SD. Data were analyzed using unpaired *t*-test to compare results between the 2 groups (SP600125 and control) in most in vitro and in vivo experiments. One-way ANOVA was used to analyze the results of RT-qPCR. *p*-Value <0.05 was considered statistically significant.

## 3. Results

### 3.1. Dkk-1 Secretion

Secretion of human Dkk-1 by Ace-1-Dkk-1 cells was confirmed by ELISA. Ace-1-Vector cells had no detectable Dkk-1 secretion, while Ace-1-Dkk-1 medium contained approximately 4.8 ng/mL after 24 h of culture.

### 3.2. Immunohistochemistry

**Figure 1:**
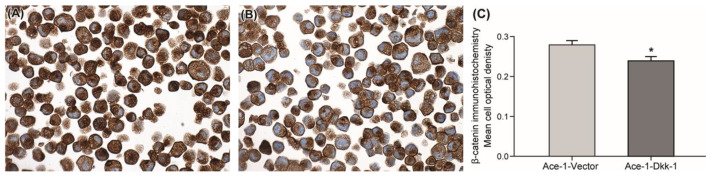
β-catenin immunohistochemical staining and quantification in Ace-1-Vector and Ace-1-Dkk-1 cells. Photomicrograph of Ace-1-Vector (**A**) and Ace-1-Dkk-1 (**B**) cells showing predominantly cytoplasmic β-catenin immunolabeling (100X, DAB/hematoxylin). (**C**) Quantification of β-catenin immunohistochemical staining over 5 fields of Ace-1-Vector and Ace-1-Dkk-1 paraffin-embedded cell pellets. Ace-1-Vector cells have a higher mean total cell optical density compared with Ace-1-Dkk-1 (* *p* < 0.05).

For both cell lines, β-catenin immunostaining was primarily within the cytoplasm (Figure 1A,B). Ace-1-Dkk-1 had significantly lower mean cell optical density of β-catenin immunostaining compared with Ace-1-Vector (Figure 1C).

### 3.3. AP-1 Reporter Activity

**Figure 2:**
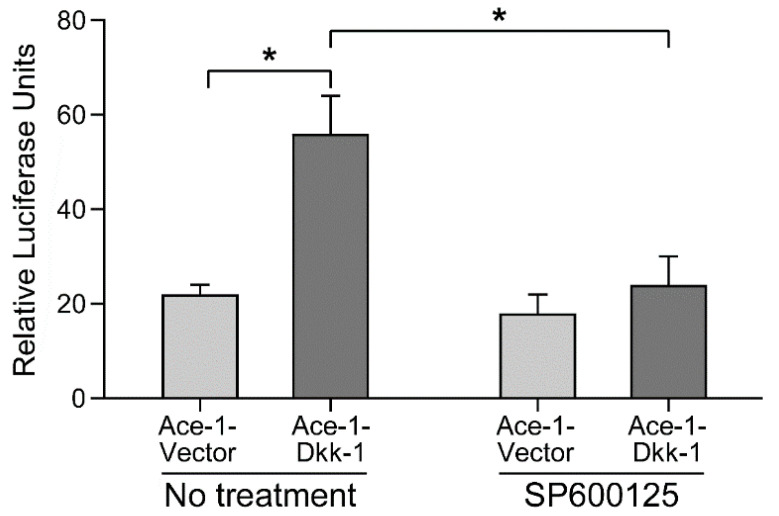
Activator protein-1 (AP-1) luciferase reporter activity of Ace-1-Vector and Ace-1-Dkk-1 cells with and without SP600125 treatment. Basal AP-1 reporter activity was markedly higher in the Ace-1-Dkk-1 cells compared with the Ace-1-Vector cells (*n* = 3, *p* < 0.0001), indicating a higher level of basal Wnt/JNK signaling. Treatment with SP600125 resulted in a significant decrease in AP-1 reporter activity in the Ace-1-Dkk-1 cells (*n* = 3, * *p* < 0.0001).

Ace-1-Dkk-1 cells had approximately a threefold increase in AP-1 reporter activity compared with Ace-1-Vector cells. Treatment with the selective JNK inhibitor, SP600125, reduced the AP-1 reporter activity of Ace-1-Dkk-1 cells almost to the Ace-1-Vector level (*p* < 0.0001). For the Ace-1-Vector cells, treatment did not result in significant changes in reporter activity (Figure 2).

### 3.4. Cell Proliferation Assay

**Figure 3:**
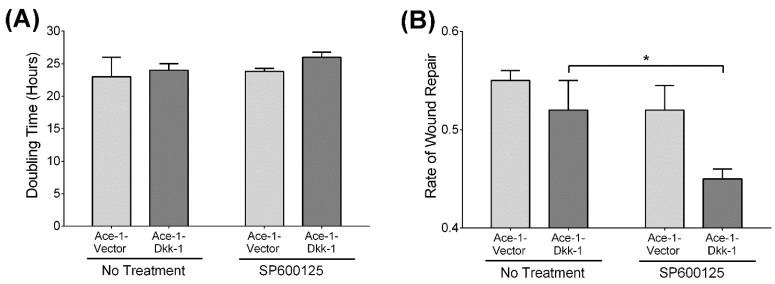
Comparison of cell proliferation and migration between Ace-1-Vector and Ace-1-Dkk-1 cells (*n* = 3, repeated twice). (**A**) Ace-1-Vector and Ace-1-Dkk-1 doubling times with and without SP600125 treatment. Basal cell proliferation was not significantly different between the two cell lines. There was no change in Ace-1-Dkk-1 proliferation after SP600125 treatment. (**B**) Basal rates of wound closure in Ace-1-Vector and Ace-1-Dkk-1 cells were similar. SP600125 significantly decreased migration only in the Ace-1-Dkk-1 cells (* *p* < 0.05).

Proliferation of Ace-1-Vector and Ace-1-Dkk-1 cells was similar in vitro (Figure 3A). No significant change in proliferation was seen in either Ace-1-Vector or Ace-1-Dkk-1 cells after treatment with SP600125.

### 3.5. Wound Healing Assay

Untreated Ace-1-Vector and Ace-1-Dkk-1 cells had similar rates of wound healing in vitro (Figure 3B). However, treatment with SP600125 reduced Ace-1-Dkk-1 migration by approximately 67% (*p* < 0.05), but had no effect on the rate of migration for Ace-1-Vector cells.

### 3.6. JNK Inhibitor (SP600125) Treatment In Vitro (RT-qPCR)

**Figure 4:**
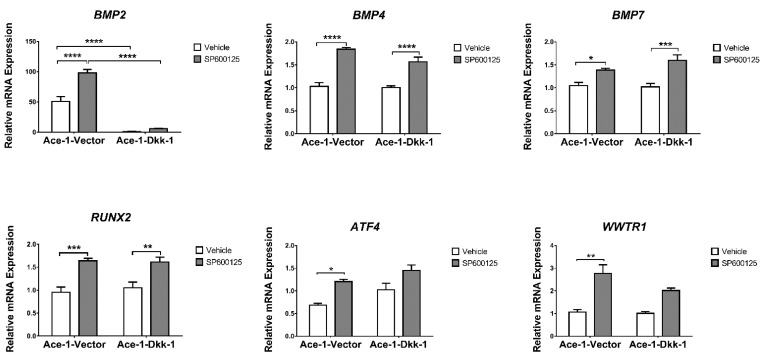
Relative in vitro mRNA expression of *BMP2*, *BMP4*, *BMP7*, *RUNX2*, *ATF4*, and *WWTR1* normalized to *GAPDH* in Ace-1-Vector and Ace-1-Dkk-1 cell lines with either vehicle or SP600125 (20 μM) treatment, *n* = 3, repeated 3 times. Significant differences between different groups are indicated with asterisks. * *p* < 0.05; ** *p* < 0.01; *** *p* < 0.001; **** *p* < 0.0001.

**Figure 5:**
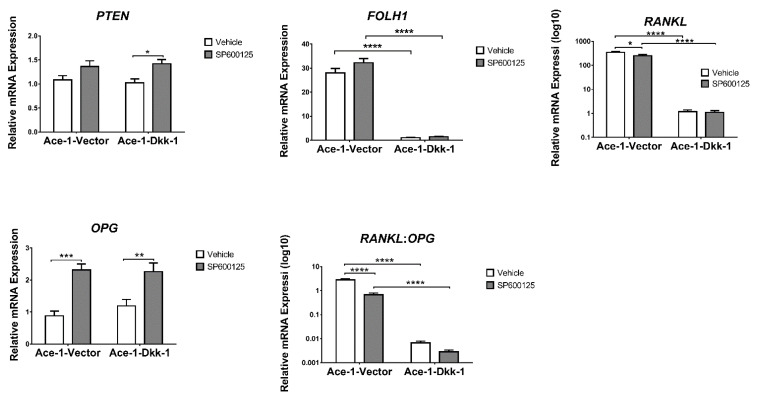
Relative in vitro mRNA expression of *PTEN*, *FOLH1*, *RANKL*, *OPG*, and *RANKL*/*OPG* normalized to *GAPDH* in Ace-1-Vector and Ace-1-Dkk-1 cell lines with either vehicle or SP600125 (20 μM) treatment, *n* = 3, repeated three times. Significant differences between different groups are indicated with asterisks. * *p* < 0.05; ** *p* < 0.01; *** *p* < 0.001; **** *p* < 0.0001.

The expression of 15 bone-related genes *(BMP2*, *BMP4*, *BMP7*, *RUNX2*, *RANKL*, *OPG*, *ATF4*, *ALDH1A1*, *WNT5A*, *WWTR1*, *MAP2K7*, *TP53*, *PTEN*, *FOLH1*, and *TCF4)* was measured by RT-qPCR in both Ace-1-Vector and Ace-1-Dkk-1 cells treated for 24 h with either SP600125 or DMSO. SP600125 significantly increased the relative mRNA expression levels of *BMP4*, *BMP7*, *RUNX2*, and *OPG* in both Ace-1-Dkk-1 and Ace-1-Vector cells and significantly increased the *PTEN* mRNA level in Ace-1-Dkk-1 cells (Figure 4 and Figure 5). SP600125 significantly increased *BMP2*, *ATF4*, and *WWTR1* in Ace-1-Vector cells (Figure 4). SP600125 significantly decreased *RANKL* in Ace-1-Vector (Figure 5) but had no effect on *ALDH1A1*, *TP53*, *WNT5A*, *FOLH1*, *TCF4*, and *MAP2K7* mRNA expression levels (data not shown). *RANKL*/*OPG* mRNA expression ratio, an indicator of osteolytic bone resorption, was significantly decreased after treatment with SP600125 in Ace-1-Vector cells (Figure 5). Dkk-1 markedly decreased the relative mRNA levels of *BMP2*, *RANKL*, *FOLH1*, and *RANKL*/*OPG* ratio in Ace-1-Dkk-1 cells compared with Ace-1-Vector cells (Figure 4 and Figure 5).

### 3.7. Subcutaneous Injection, JNK Inhibitor (SP600125) Treatment, and RT-qPCR

**Figure 6:**
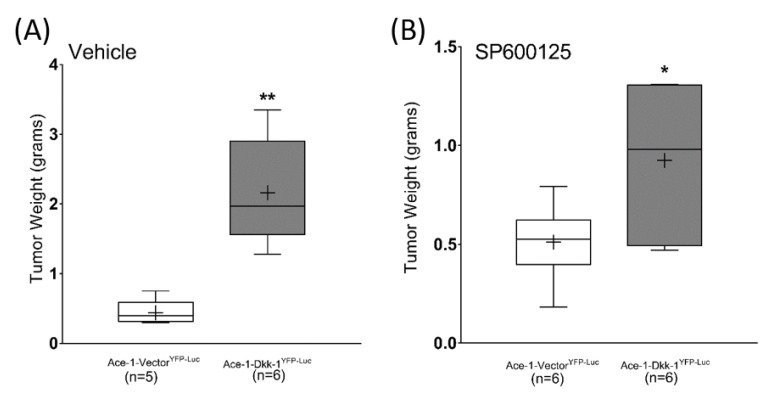
Average tumor weight in mice bearing Ace-1-Dkk-1^YFP-LUC^ xenografts (*n* = 12) was significantly greater compared with mice bearing Ace-1-Vector^YFP-LUC^ xenografts (*n* = 11). (**A**) Control (Vehicle) mice. (**B**) SP600125-treated mice. * *p* < 0.05; ** *p* < 0.001.

Subcutaneous injections were performed with Ace-1-Dkk-1^YFP-Luc^ or Ace-1-Vector^YFP-Luc^ cells in athymic mice to measure the effects of Dkk-1 and SP600125 on tumor growth. Dkk-1 increased tumor growth in Ace-1-Dkk-1^YFP-LUC^ xenografts compared with Ace-1-Vector^YFP-LUC^ xenografts. The average tumor weight in mice bearing Ace-1-Dkk-1^YFP-LUC^ tumors (mean = 2.16 gms ± 0.32) was significantly greater compared with tumors with Ace-1-Vector ^YFP-LUC^ (mean = 0.44 gms ± 0.08) (Figure 6)

**Figure 7:**
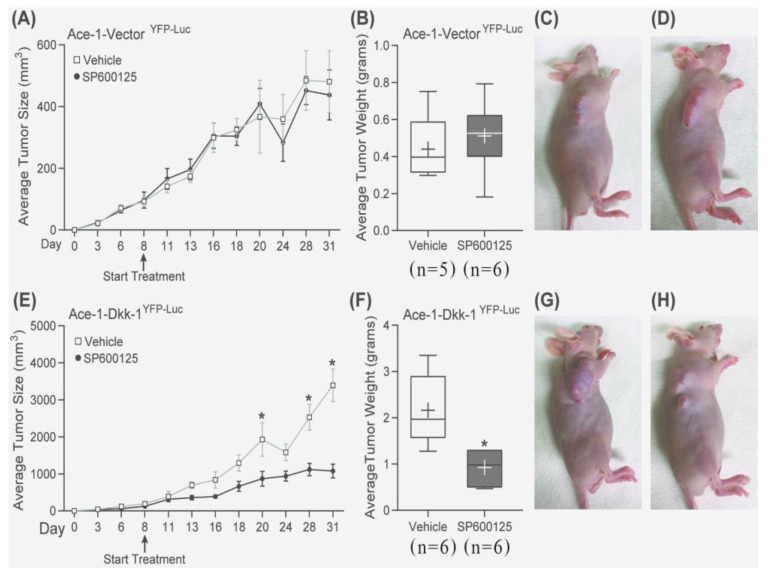
Growth curves, average tumor weight, and tumor sizes of mice bearing Ace-1-Vector^YFP-LUC^ and Ace-1-Dkk-1^YFP-LUC^ subcutaneous xenografts with and without SP600125 treatment were compared. (**A**) Growth curves of Ace-1-Vector^YFP-LUC^ subcutaneous xenografts (both control (*n* = 5) and SP600125 treated (*n* = 6)) in nude mice. (**B**) Average tumor weight in mice bearing Ace-1-Vector^YFP-LUC^ xenografts (both control (*n* = 5) and SP600125 treated (*n* = 6)). (**C**) Control mice bearing Ace-1-Vector^YFP-LUC^ xenografts. (**D**) SP600125-treated mice bearing Ace-1-Vector^YFP-LUC^ xenografts. (**E**) Growth curves of Ace-1-Dkk-1^YFP-LUC^ subcutaneous xenografts (both control (*n* = 6)) and SP600125 treated (*n* = 6)) in nude mice. (**F**) Average tumor weight in mice bearing Ace-1-Dkk-1^YFP-LUC^ xenografts (both control (*n* = 6) and SP600125 treated (*n* = 6)). (**G**) Control mice bearing Ace-1-Dkk-1^YFP-LUC^ xenografts. (**H**) SP600125-treated mice bearing Ace-1-Dkk-1^YFP-LUC^ xenografts. SP600125 significantly decreased tumor growth in mice bearing Ace-1-Dkk-1^YFP-Luc^, but not in mice bearing Ace-1-Vector^YFP-Luc^ xenografts. * *p* < 0.05.

Interestingly, SP600125 significantly decreased the growth-promoting effect of Dkk-1 in mice bearing Ace-1-Dkk-1^YFP-LUC^ tumors (treatment started on day 8 after injection) compared with mice with Ace-1-Vector tumors (Figure 7A–H). 

**Figure 8:**
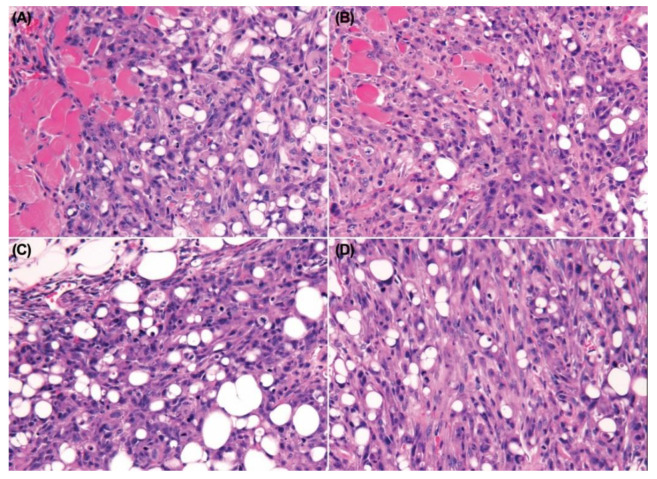
Canine prostate carcinoma, nude mouse subcutaneous xenografts. (**A**) Ace-1-Vector^YFP-LUC^ with vehicle. (**B**) Ace-1-Vector^YFP-LUC^ with SP600125. (**C**) Ace-1-Dkk-1^YFP-LUC^ with vehicle. (**D**) Ace-1-Dkk-1^YFP-LUC^ with SP600125. The xenograft tumor cells had an increased spindle cell morphology after treatment with SP600125 in both Ace-1-Vector^YFP-LUC^ and Ace-1-Dkk-1^YFP-LUC^ xenografts. Hematoxylin and eosin (20×, hematoxylin and eosin (HE)).

The histopathology of SP600125-treated tumor cells had a greater spindle cell appearance compared with nontreated tumors. The neoplastic cells lost their epithelial phenotype (polygonal-shaped cells) and gained a mesenchymal phenotype (spindle-shaped cells) after treatment with SP600125 in both Ace-1-Vector^YFP-LUC^ and Ace-1-Dkk-1^YFP-LUC^ xenografts (Figure 8A–D).

**Figure 9:**
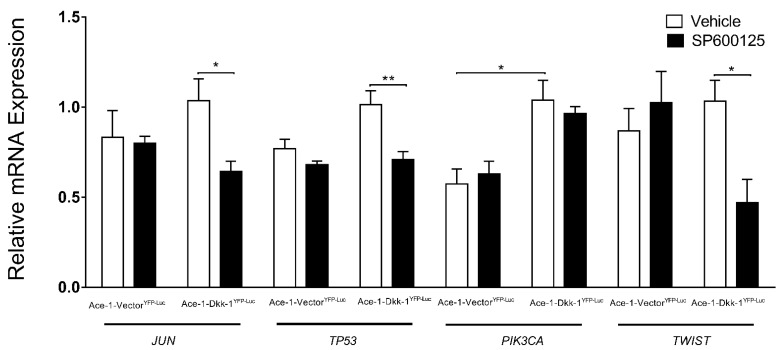
Relative in vivo mRNA expression of *JUN*, *TP53*, *PIK3CA*, and *TWIST* normalized to *GAPDH* in Ace-1-Vector^YFP-LUC^ and Ace-1-Dkk-1^YFP-LUC^ xenografts with either vehicle or SP600125 (5 mg/Kg). Significant differences between different groups are indicated with asterisks. * *p* < 0.05; ** *p* < 0.001.

The expression of 17 JNK-related genes that play a role in PCa progression and bone metastasis *(JUN*, *FOS*, *TP53*, *PTEN*, *PIK3CA*, *TWIST*, *AKT1*, *ALDH1A1*, *ATF4*, *BMP2*, *MAPK9*, *OPG*, *RANKL*, *PTHrP*, *RUNX2*, *TGFβ*, and *Wnt11)* was measured by RT-qPCR in mice xenografts. SP600125 significantly decreased the mRNA expression levels of *JUN*, *TP53*, *and TWIST* in treated Ace-1-Dkk-1^YFP-LUC^ xenografts compared with Ace-1-Dkk-1^YFP-LUC^ xenografts with vehicle (Figure 9). SP600125 had no effect on *FOS*, *PTEN*, *PIK3CA*, *AKT1*, *ALDH1A1*, *ATF4*, *BMP2*, *MAPK9*, *OPG*, *RANKL*, *PTHrP*, *RUNX2*, *TGFβ*, and *Wnt11* (Supplementary Figures S1 and S2). Dkk-1 significantly increased *PIK3CA* in Ace-1-Dkk-1^YFP-LUC^ xenografts compared with Ace-1-Vector^YFP-LUC^ xenografts (Figure 9).

### 3.8. Intratibial Injection and JNK Inhibitor (SP600125) Treatment

**Figure 10:**
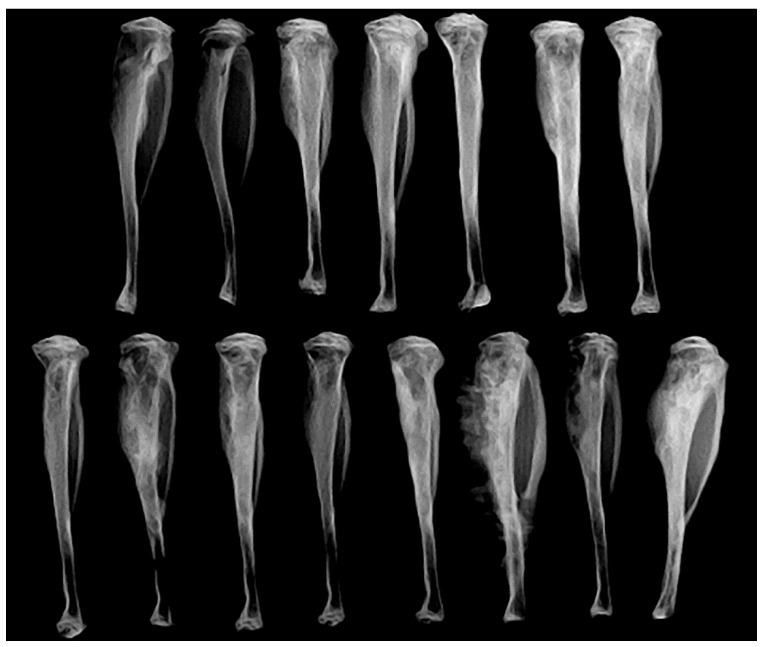
Faxitron radiographic images of Ace-1-Dkk-1^YFP-LUC^ intratibial bone tumors in control (top panel) and SP600125-treated mice (bottom panel). The SP600125-treated mice have markedly increased intramedullary and periosteal new bone formation compared with control mice. This is especially evident in the periosteum, as demonstrated by irregularly shaped tumors compared with the smooth periosteal margins in control mice.

Intratibial injections were performed with Ace-1-Dkk-1^YFP-Luc^ cells to evaluate the effect of SP600125 on tumor growth in the bone. The growth of bone tumors was measured by bioluminescent imaging on days 7 and 14 after tumor cell transplantation. There was no difference in bioluminescence between SP600125-treated mice and control mice (data not shown), demonstrating similar growth of viable tumor cells. However, the radiographs between the two groups had marked differences. The control tumors had smooth external bone surfaces with minimal periosteal new bone and intramedullary new bone formation with mild to moderate bone lysis. Bone tumors from mice treated with SP600125 had irregular shapes with dense intramedullary bone formation (increased radio-opacity), abundant periosteal new bone formation in the tibias and fibulas, and large foci of bone lysis (Figure 10).

**Figure 11:**
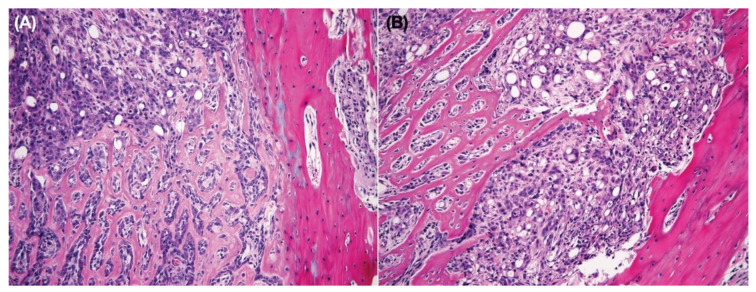
Tibia. Nude mouse. (**A**) Ace-1-Dkk-1^YFP-LUC^ with vehicle. (**B**) Ace-1-Dkk-1^YFP-LUC^ with SP600125. Cortical bone is on the left. The Ace-1 prostate cancer cells replaced the marrow with invasive cancer cells and abundant new woven bone formation (10×, HE).

Histopathology confirmed the radiographic findings. Both groups of mice had growth of well-differentiated PCa cells with both osteoclastic bone lysis and new medullary woven bone. The SP600125-treated tumors were interpreted to be more invasive with cortical bone lysis and secondary periosteal new bone formation. SP600125 also may have induced the formation of a denser medullary bone. The tumor cells in both groups grew in the diaphyseal regions, replacing most of the bone marrow (Figure 11A,B).

## 4. Discussion

Nineteen Wnts were identified in humans and mice that bind to 10 cognate receptors from the FZD family that regulate several signaling cascades. The two best characterized are the canonical and the noncanonical Wnt signaling cascades. Canonical Wnt signaling depends on the cytoplasmic stabilization and subsequent nuclear translocation of β-catenin for the transcription of Wnt-specific genes associated with stemness, cell proliferation, differentiation, migration, and apoptosis [29,30]. The noncanonical Wnt signaling pathways are classified into either the planar cell polarity (PCP) or the Wnt-calcium pathway [31]. Previously, we showed that Dkk-1 overexpression in Ace-1 cells activated the noncanonical Wnt/JNK signaling pathway, resulting in increased tumor growth and the incidence of bone metastasis [5]. Ace-1 cells are a relevant model of canine prostate cancer, as they form localized and metastatic prostate cancer in immunosuppressed beagle [29], and left ventricular injection of Ace-1 cells in rodent results in almost exclusive metastasis to the bone [32]. This cell line has also been used in dogs as a model for developing therapeutic strategies for human prostate cancer patients [33]. In the present study, we reported increased Wnt/JNK signaling in Ace-1-Dkk-1 cells and showed that Wnt/JNK signaling resulted in increased tumor growth.

The canonical pathway is initiated by the binding of Wnt ligands to the FZD receptor and LRP5/6 to activate signaling through β-catenin [34]. While the noncanonical Wnt/JNK pathway is less understood, it has been reported that Wnt ligands also bind to the FZD receptor in the absence of LRP5/6, resulting in phosphorylation of disheveled (Dsh) and JNK activation [35]. In the present study, Dkk-1 was secreted from the Ace-1-Dkk-1 cells and significantly decreased β-catenin compared with Ace-1-Vector cells. Moreover, Dkk-1 significantly increased AP-1 activity, a downstream product of the noncanonical Wnt/JNK pathway, in Ace-1-Dkk-1 compared with Ace-1-Vector cells. Thus, the effect of Dkk-1 in inhibiting canonical Wnt and activating noncanonical Wnt/JNK signaling pathways was demonstrated. Blocking noncanonical Wnt/JNK using a JNK inhibitor (SP600125) markedly decreased the AP-1 activity in Ace-1-Dkk-1, but not in Ace-1-Vector cells, suggesting that this pathway is not active in the Ace-1-Vector cells.

The expression of BMPs by PCa was reported to induce osteoblast differentiation through both the canonical (Wnt/β-catenin) and noncanonical (Wnt/JNK) signaling pathways in C4-2B cells (PCa cell line), and Dkk-1 blocked the osteoblastic differentiation in vitro [36]. Dkk-1 was highly expressed in breast cancer patients who predominantly developed osteolytic bone metastases [34,37]. Dkk-1 has been shown to induce osteolysis and inhibit osteoblast differentiation in PCa bone metastases by inhibiting the Wnt signaling pathway. Moreover, Dkk-1 decreased the osteoblastic differentiation and mineralization in PCa cell lines in vitro and has the ability to switch the bone metastasis phenotype from osteoblastic to osteolytic [38]. In this study, Dkk-1 significantly downregulated *BMP2* expression in vitro; therefore, we speculated that the osteolytic appearance of Ace-1-Dkk-1 arises from the *BMP2* inhibition effect of Dkk-1.

The expressions of genes associated with osteoblast differentiation, including *BMP4*, *BMP7*, *RUNX2*, and *OPG*, in both Ace-1-Dkk-1 and Ace-1-Vector were investigated in vitro. *BMP4* was reported to induce osteoblast differentiation in PCa-118b (PCa cell line) [39]. *BMP7* was highly expressed in metastatic bone lesions of prostate cancer, and expression was related to osteoblastic metastasis [40]. Moreover, *RUNX2* upregulation was associated with osteoblast differentiation through the canonical Wnt pathway [41]. *OPG* is a decoy receptor expressed by osteoblast that inhibits the binding of *RANKL* on osteoblast to its receptor RANK on osteoclast and negatively regulates osteoclast differentiation and reduces bone resorption [7]. We found that SP600125 upregulated all of these genes in vitro. These results indicated that SP600125 restored the canonical Wnt signaling in Ace-1-Dkk-1 cells.

The upregulation of cell-survival-related genes, including *ATF4* and *WWTR1,* was demonstrated in vitro in Ace-1-Vector cells with SP600125 treatment. *ATF4* expression is upregulated by tumor microenvironment stress, including hypoxia and nutrient deprivation. This gene promotes cancer cell adaptation to such conditions by transcriptionally upregulating genes essential for redox balance, angiogenesis, and autophagy. Moreover, *ATF4* has been implicated in cancer progression and drug resistance [42]. *WWTR1* is a downstream transcriptional coactivator of the Hippo pathway and has been reported to be overexpressed in various human cancers, which correlated to cancer aggressiveness by promoting malignant cell proliferation and inhibiting apoptosis [43]. Our data demonstrated that SP600125 upregulated the in vitro expression of these genes in Ace-1-Vector cells, which could enhance tumor cell survival.

The upregulation of *PTEN* expression in Ace-Dkk-1 cells was also observed in this study. *PTEN* is a tumor suppressor gene that is inactivated by mutation or deletion in advanced PCa. *PTEN* exerts its tumor suppressor function by inducing cell cycle arrest through inhibition of the PI3K signaling pathway [44]. JNK overexpression was found to downregulate *PTEN* expression. Decreased *PTEN* expression promotes cell proliferation, decreases apoptosis, and enhances tumor angiogenesis [45]. In the present study, SP600125 upregulated *PTEN* expression in Ace-1-Dkk-1 cells in vitro, which may be due to JNK inhibition.

Interestingly, Dkk-1 downregulated the in vitro expression of *FOLH1* in Ace-1-Dkk-1 cells. Low-to-moderate expression of *FOLH1*, a prostate-specific membrane antigen (PSMA), was observed in osteoblastic activity [46]. *FOLH1* was reported to be upregulated in PCa, and its expression has been correlated with cancer aggressiveness. Moreover, *FOLH1* may be a potential marker for PCa therapeutics [47]. In men with PCa, high *FOLH1* expression has been correlated with low androgen expression [48]. Most canine prostate cancers are discovered at a late stage in progression and are typically androgen independent [49,50]. Consequently, the decrease in the expression of *FOLH1* in Ace-1-Dkk-1 cells could alter the phenotype of bone metastases from osteoblastic to osteolytic.

Dkk-1 significantly upregulated *PIK3CA* in Ace-1-Dkk-1^YFP-LUC^ xenografts compared with Ace-1-Vector^YFP-LUC^ xenografts. Oncogenic activation of the phosphatidylinositol-3-kinase (PI3K) pathway is a common event in PCa that promotes tumorigenesis, disease progression, and therapeutic resistance [51]. In addition, genetic alterations of the PI3K pathway are common in PCa patients with up to 42% of primary versus 100% of metastatic prostatic tumor samples [51,52]. Dkk-1 contributed to tumor growth through the regulation of Wnt signaling and PI3K/AKT signaling in cancer cells [53]. Therefore, it is possible that the increased tumor growth in Ace-1-Dkk-1^YFP-LUC^ xenografts was related to the upregulated expression of *PIK3CA.*

There was no difference in in vitro cell proliferation between Ace-1-Vector and Ace-1-Dkk-1 cells. Therefore, we speculate that the increased in vivo subcutaneous growth in Ace-1-Dkk-1^YFP-LUC^ compared with Ace-1-Vector^YFP-LUC^ xenografts was due to a paracrine interaction with the tumor microenvironment, which is consistent with a previous study [4]. However, SP600125 reduced the tumor growth of Ace-1-Dkk-1^YFP-LUC^ but not Ace-1-Vector^YFP-LUC^ xenografts. SP600125 treatment led to the acquisition of a spindle-shaped tumor cell appearance in both Ace-1-Dkk-1^YFP-LUC^ and Ace-1-Vector^YFP-LUC^ xenografts. This was suggestive of an EMT phenotype that should be confirmed. It may be therapeutically advantageous to use SP600125 in combination with other drugs in the early stages of PCa.

In vivo, SP600125 downregulated *JUN*, *TP53*, and *TWIST* expressions in Ace-1-Dkk-1^YFP-LUC^ xenografts. *JUN* was identified as a master regulator gene in cell proliferation, differentiation, and apoptosis [54]. *JUN* overexpression induced oncogenic transformation, increased tumor formation, and invasion in human breast cancer cells [55]. The *TP53* tumor suppressor gene is mutated (30%) in PCa, and *TP53* mutations were associated with tumor progression in PCa [56,57]. A previous study revealed *PTEN* loss concurrently with *TP53* structural rearrangements in PCa cases [58]. The levels of *TP53* expression and types of *TP53* mutation were found to be closely related to the prognosis in triple-negative breast cancer. Low *TP53* expression in missense mutation patients and high *TP53* expression in *TP53* deletion mutation patients were associated with poor prognosis [59]. In this study, we found that SP600125 downregulated *TP53* expression in Ace-1-Dkk-1^YPF-LUC^ xenografts and reduced tumor sizes in vivo. Low *TP53* expression is not always a negative prognostic indicator, and its prognostic values can be varied on *TP53* mutation types. Thus, *TP53* mutation in PCa should be further investigated. *TWIST* is an important gene in cancer metastasis due to its contribution in EMT, angiogenesis, and chromosomal instability [60,61], and its downregulation is commonly associated with decreased metastasis [62].

Our previous work showed that Dkk-1 inhibited the canonical Wnt-induced osteoblastic bone metastases [4]. In the current study, SP600125 did not alter tumor growth in the bone but increased the Ace-1-Dkk-1^YFP-Luc^ osteoblastic and osteolytic phenotype of the bone tumors with increased cortical invasion and secondary induction of periosteal new bone formation. The intratibial woven bone formation induced by SP600125 through the inhibition of Wnt/JNK signaling and the activation of the canonical Wnt signaling indicates that the bone microenvironment plays a pivotal role in new bone formation through a paracrine mechanism.

## 5. Conclusions

Overexpression of human Dkk-1 increased tumor growth in vivo and upregulated the noncanonical Wnt/JNK signaling pathway in Ace-1-Dkk-1 cells, resulting in downstream alterations in gene expression involved in the osteoblast differentiation, cell proliferation, and microscopic appearance of the cells. Thus, Ace-1-Vector and Ace-1-Dkk-1 cells are useful models for studying the biological and molecular characteristics of canonical Wnt and noncanonical Wnt/JNK signaling pathways in PCa, respectively. In addition, SP600125 could be an alternative adjuvant therapy for decreasing tumor size in dogs and humans with spontaneous PCa that expresses high levels of Dkk-1. However, SP600125 may have the potential to increase the local invasiveness of bone metastases, and the phenotype and drug responsiveness of the tumor cells may depend on the local microenvironment.

## Data Availability

The data presented in this study are available on request from the corresponding author.

## Supplementary Materials

The following are available online at https://www.mdpi.com/article/10.3390/vetsci8080153/s1, Figure S1: Relative in vivo mRNA expression of *FOS, PTEN, PIK3CA, AKT1, ALDH1A1, ATF4, BMP2* and *MAPK9* normalized to normalized to *GAPDH* in Ace-1-Vector^YFP-LUC^ and Ace-1-Dkk-1^YFP-LUC^ xenografts with either vehicle or SP600125 (5 mg/Kg), Figure S2: Relative in vivo mRNA expression of *OPG, RANKL, PTHrP, RUNX2, TGFβ,* and *WNT11* normalized to normalized to *GAPDH* in Ace-1-Vector^YFP-LUC^ and Ace-1-Dkk-1^YFP-LUC^ xenografts with either vehicle or SP600125 (5 mg/Kg), Table S1: Primer used for RT-qPCR experiments.
