# Supplementary material for: Effect of Dickkopf-1 (Dkk-1) and SP600125, a JNK Inhibitor, on Wnt Signaling in Canine Prostate Cancer Growth and Bone Metastases
Source: Vet Sci. 2021 Aug 1;8(8):153. doi: 10.3390/vetsci8080153 (PMC8402794; doi:10.3390/vetsci8080153)
Supplement: Supplementary file 1 [file vetsci-08-00153-s001.zip › vetsci-1273609-supplement/vetsci-1273609-Supplemental figures.pdf]

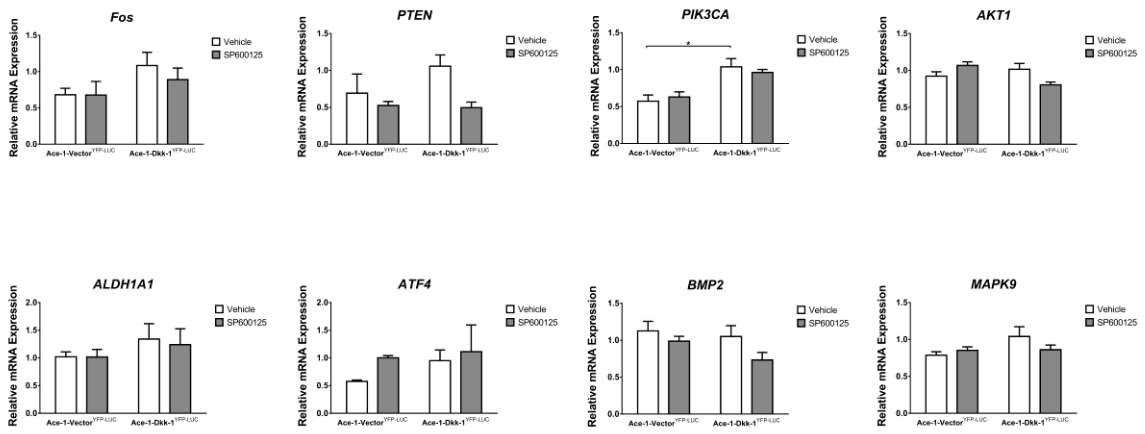

Figure S1: Relative *in vivo* mRNA expression of *FOS, PTEN, PIK3CA, AKT1, ALDH1A1, ATF4, BMP2* and *MAPK9* normalized to normalized to *GAPDH* in Ace-1-Vector YFP-LUC and Ace-1-Dkk-1 YFP-LUC xenografts with either vehicle or SP600125 (5 mg/Kg). Significant differences between different groups are indicated with asterisks.

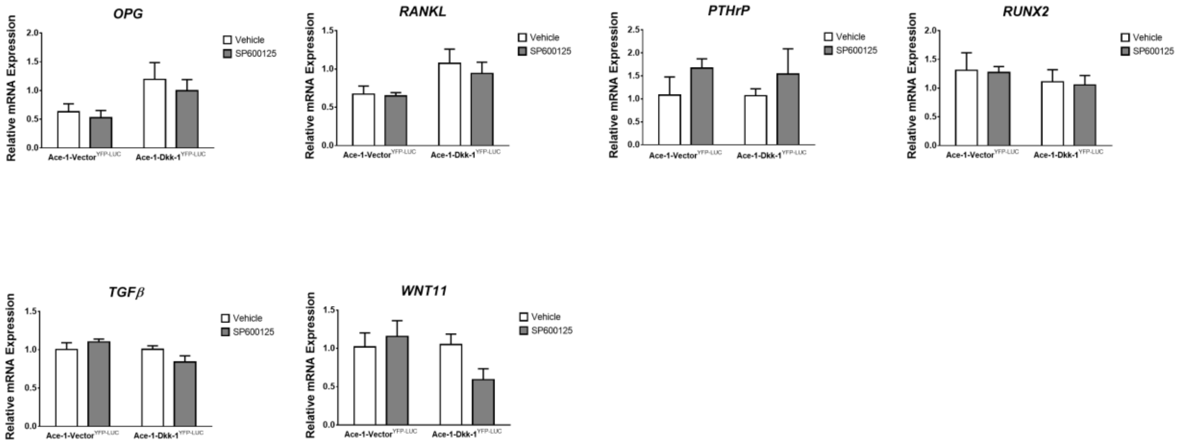

Figure S2: Relative *in vivo* mRNA expression of *OPG, RANKL, PTHrP, RUNX2, TGFβ,* and *WNT11* normalized to normalized to *GAPDH* in Ace-1-Vector YFP-LUC and Ace-1-Dkk-1 YFP-LUC xenografts with either vehicle or SP600125 (5 mg/Kg). Significant differences between different groups are indicated with asterisks.
